# Facilitators and challenges to access fresh fruits and vegetables in a low to middle income group in Bangladesh: Consumers’ perception

**DOI:** 10.1016/j.puhip.2026.100740

**Published:** 2026-01-30

**Authors:** Fariza Fieroze, Md Badruddin Saify, Ummey Farwah, Rumana Huque

**Affiliations:** a ARK Foundation, Suite-A1, C3-C4, House No.06, Road No.109, Gulshan-2, Dhaka, Bangladesh; b Department of Economics, University of Dhaka, Dhaka, Bangladesh

**Keywords:** Vegetables, Fruits, Fresh, Facilitators, Challenges, Consumer's perception, Bangladesh

## Abstract

**Objective:**

To explore challenges and barriers faced by vulnerable women and men in accessing fresh foods, and how they overcome existing barriers to healthier dietary improvements in households and communities.

**Study design:**

This study employed an explorative qualitative research design.

**Methods:**

Using purposive sampling through a local primary healthcare facility, 16 in-depth interviews (8 male, 8 female household heads) and 2 focus group discussions (8 participants each, gender-segregated) were conducted with vulnerable slum populations in Mirpur, Dhaka, Bangladesh, between September 2020 and August 2021. Participants were low-to-middle income household heads responsible for grocery shopping and menu decisions. Data were collected in the local language-Bangla, audio-recorded, transcribed verbatim, and analyzed using thematic framework analysis.

**Results:**

Key findings identified price fluctuations as a major factor impacting purchase quantity and consumption. Participants knowingly purchased stale items due to necessity, while personal food preferences often dominated healthy food choices. Limited accessibility to wholesale markets forced dependence on mobile vendors and small shops regardless of product freshness. Furthermore, product availability fluctuated with natural calamities, the pandemic, and seasonal effects, alongside intentional crises created by market intermediaries. Conversely, knowledge of healthy foods helped determine optimal family menus.

**Conclusions:**

Given the rising non-communicable disease burden in Bangladesh's urban areas, swift accessibility to fresh fruits and vegetables is vital for increasing healthy dietary intake among low-to-middle income populations.

## 1 What this study adds

• First qualitative study of consumer perceptions on fresh fruit and vegetable access among urban low-to-middle income populations in Bangladesh
• Illustrates how consumers define freshness (color, firmness) and unearth crisis purchasing behaviors even accepting stale items due to necessity during COVID-19
• Provides a gender-disaggregated lens into household food purchase, decision-making, and fresh produce selection abilities

## 2 Implications for policy and practice

• Establish more community-level wholesale markets and support mobile vendors to maintain product freshness throughout the day
• Implement price monitoring, regulation, and emergency supply protocols to address fluctuations caused by disasters, pandemics, and artificial hoarding
• Combine nutrition education with structural interventions addressing affordability and supply chain barriers for effective dietary improvement

## 3 Introduction

Consuming fresh fruit and vegetables is an important element for leading a healthy life [[1], [2], [3], [4]]. Vegetables are essential sources of vitamins, micronutrients and dietary fibre [5]. Vegetables make foods more palatable, help in digestion, and provide nutrition to the human body, thus preventing malnutrition which Bangladesh has experienced as a burden recently [6,7]. Inadequate intake of fruits and vegetables is associated with poor health outcomes such as different chronic diseases and being overweight. Consuming fresh fruits and vegetables is considered important for the prevention of non-communicable diseases, i.e., heart disease, diabetes, cancer, and obesity. The current Global Burden of Disease Analysis stated that low fruit intake and insufficient vegetable intake are responsible for 4.9 million and 1.8 million deaths per year respectively.

Barriers are negative factors that inhibit the consumption of fresh fruits and vegetables [4]. Research indicates that income is one of the potential barriers to accessing healthy food choices [8]. People from lower-income groups tend to develop unhealthy eating habits due to the high cost of fruits and vegetables [9]. These income-related disparities in consuming fruits and vegetables are also observed in high income countries among people whose living standards are at or below poverty [8]. Dietary habits of urban people largely depend on processed foods because of the perishable nature of harvested crops. Though the consumption of fresh fruits and vegetables is important, the perception of freshness varies from person to person. Though there is a scarcity of data about what freshness means to consumers, it is a significant factor in consumers' selection of fruits and vegetables [10]. To keep fruits and vegetables fresh, they must be transported quickly to markets or to consumers after harvesting [11]. In Fiji, it was reported that urban populations were aware of the health benefits of fruits and vegetables but lack of accessibility and affordability was a barrier to getting those foods [3]. Several studies identified various barriers to accessing fresh vegetables and fruits such as high cost, lack of time, unpleasant taste, lack of habit, high perishability of these foods, forgetfulness, lack of satiation, difficulty in transportation to work, little availability in the market, preparation and availability of convenience foods [4,12].

Over the past decades, vegetable production in Bangladesh has been increasing. The total amount of vegetable production is 3.73 million metric tons every year, however, the annual demand is 13.25 million metric tons [7,13]. The recommended daily intake of fruits and vegetables is 400 g for an adult, however, fruits and vegetable consumption per capita per day in Bangladesh is 203.8 g [14,15].

There are different types of vegetable and fruit market systems in Bangladesh such as rural primary markets, rural assembly markets, rural secondary markets, and urban retail markets which are controlled by various actors. Although vegetables are perishable in nature, 25–40 % of vegetables are destroyed because of inappropriate handling, packaging, transportation and storage practices [7]. On one hand, farmers use harmful chemicals and toxic pesticides during the production of vegetables, and on the other hand, sellers also use preservatives and artificial colours to keep the vegetables fresh. This poses a great public health concern and makes it hard for people to get fresh vegetables in the market [13].

Comparisons across South Asian countries show both similarities and differences in fruit and vegetable consumption patterns and market access challenges. A study examining the cost of nutritious diets across Bangladesh, Bhutan, India, and Nepal found that fruit and vegetable availability varies significantly across the region, with substantial variations in consumption patterns [16]. In Bangladesh, the high cost of healthy diets is partly attributable to expensive dairy products and the substantial share of diet costs allocated to fruits and vegetables. A systematic review exploring barriers to fruit and vegetable consumption in low- and middle-income countries found multiple common barriers across South Asian countries including high costs relative to income, poor market infrastructure and logistics, limited year-round availability of diverse produce, and inadequate cold chain facilities [16,17].

The supply of fresh fruits and vegetables is plagued with several barriers. To date, most quantitative studies focus on vicinity and food types stored in the community. More research, especially qualitative research, is needed for better knowledge about barriers and access to fresh fruits and vegetables [2]. To address this gap, our study used focus group discussions and in-depth interviews to obtain an understanding of facilitators and barriers to access fruits and vegetables among low to middle-income urban people.

## 4 Methods

An explorative, qualitative study method was adopted using an inductive approach that included in-depth interviews (IDI) and focus group discussions (FGDs) to explore challenges and barriers faced by vulnerable women and men in accessing vegetables and fruits, and to identify how they overcome existing barriers to healthier dietary improvements in households and communities. As the literature on this specific demographic was scarce, no pre-existing theoretical framework was used. Instead, we allowed the themes to emerge directly from the data. A gender equity lens was used in exploring challenges and potential benefits, particularly considering women's decision-making roles in what to buy and cook, the influences of other household members and specific access barriers faced by women.

An even mix of male and female household heads from low-to-middle-income backgrounds with minimum to average education were selected. They regularly did grocery shopping for their families and had the power to decide the family menu. Purposive sampling was used with the targeted population being reached through a local primary healthcare facility to facilitate communication and avoid mistrust. The participants were residents of the Mirpur area located in Dhaka, Bangladesh. A total of 16 IDIs were conducted with 8 male and 8 female household heads. Two FGDs were also conducted, one with 8 female household heads and another with 8 male household heads.

An in-depth interview topic guide was prepared by the researchers to elicit discussion about their purchasing ability, consumption patterns, grocery shopping behaviours, perceived barriers and challenges to accessing fresh vegetables and fruits, and the strategies to improve accessibility in their area. The FGD topic guide was also similar to the IDI topic guide to explore the same issues from the group discussion. The topic guide was piloted with one female household head to refine it. The consent form and information sheet were also prepared to obtain consent from the participants for their participation in the study.

All the qualitative data were collected between September 2020 and August 2021. At the time of recruitment, an information sheet was provided to each participant and written consent was obtained. The verbal consent was recorded before the telephone interview started. All the participants completed a demographic survey.

All the qualitative data were collected in the local language in a room at the primary healthcare facility. All the FGDs and interviews were audio recorded. In consideration of the pandemic situation, every possible precautionary measure and social distancing were well maintained following national COVID-19 safety guidelines.

Considering the availability of the participants, 6 interviews were conducted over the telephone and 10 were conducted face to face. Two experienced qualitative researchers conducted the interviews. The interviews lasted 25–50 min.

FGDs were moderated face to face by an experienced qualitative researcher while another researcher took notes and assisted the moderator. The duration of the FGDs was 1 h 10 min to 1 h 17 min. Some questions were probed repeatedly to obtain new information. The sample size of 16 interviews and 2 focus group discussions was predetermined based on the study design and resource constraints. However, data saturation was monitored during the analysis phase. Thematic analysis showed consistent patterns across participants after the 12th interview, with the remaining data confirming existing themes rather than introducing new concepts. Thus, this approach suggested adequate sample size for saturation.

The interviews were transcribed verbatim and the accuracy was checked by the researchers by reading the transcripts against the audio and translating them into English. A thematic framework was developed by two experienced qualitative researchers. The data were analyzed using Microsoft Excel 2013 software following the developed framework [18].

The thematic framework was developed in English based on the research questions and the topic guides. The framework was then piloted with two transcripts by two researchers, one transcript by each before finalizing the framework. It was then systematically applied to the qualitative data. The overall analysis was done by three researchers. Response summaries from the participants were prepared and the quotations were entered. Descriptive findings were written by reviewing and interrogating the charted data.

## 5 Results

Participants’ characteristics are shown in Table 1, Table 2. All the participants did the grocery shopping for their families from the local vendors such as carts or local wet markets. During the data analysis, 8 major common themes were identified as barriers as well as the facilitators of accessing and consuming fresh fruits and vegetables. These were: 1. Price, 2. Necessity, 3. Personal choice, 4. Availability, 5. Accessibility, 6. Knowledge, 7. Food adulteration, and 8. Income.

**Table 1:** The in-depth- interview participants’ characteristics.

Participant ID	Age	Sex	Family Members	Education	Occupation	Total Earning Person	Income
*HD*^a^_DK^b^_F^c^*01*	38	Female	6	No education	Housewife	1	20000–25000
*HD_DK_F02*	30	Female	6	Class 5	Housemaid	2	20000
*HD_DK_F03*	40	Female	4	Class 8	Office assistant	2	10000–15000
*HD_DK_F04*	50	Female	5	Class 9	Businesswoman	2	20000
*HD_DK_F05*	38	Female	6	Class 5	Housemaid	2	10000
*HD_DK_F06*	30	Female	4	Class 5	Garment worker	4	21000
*HD_DK_F07*	35	Female	4	Class 5	Housewife	1	15000
*HD_DK_F08*	35	Female	2	SSC^d^	Business	1	10000–15000
*HD_DK_*M^e^01	30	Male	4	Masters	Service holder	1	15000
*HD_DK_M02*	40	Male	4	Diploma	Lab technician	3	30000
*HD_DK_M03*	25	Male	2	SSC	Office assistant	1	10000
*HD_DK_M04*	56	Male	4	HSC^f^	Business	1	15000
*HD_DK_M05*	36	Male	6	BA	Service holder	1	25000
*HD_DK_M06*	41	Male	4	Masters	Service holder	2	30000
*HD_DK_M07*	45	Male	5	Class 7	Job	2	14500
*HD_DK_M08*	75	Male	7	Class 3	Job	4	40000

a Healthy Diet.

b Dhaka.

c Female.

d Secondary School Certificate.

e Male.

f Higher School Certificate.

**Table 2:** The focus group discussion participants’ characteristics.

Participants ID	Age	Sex	Family members	Education	Occupation	Total earning person	Income
*FGD1_HD_M01*	26	Male	5	BSC	Service holder	1	20000
*FGD1_HD_M02*	28	Male	4	Class 5	Business	1	15000
*FGD1_HD_M03*	42	Male	4	Class 5	Job	1	10000
*FGD1_HD_M04*	47	Male	5	SSC	Job	1	10000
*FGD1_HD_M05*	50	Male	4	Class 5	Driver	1	15000
*FGD1_HD_M06*	43	Male	4	SSC	Job	1	10000
*FGD1_HD_M07*	51	Male	4	Class 7	Driver	1	10000
*FGD1_HD_M08*	50	Male	4	Class 3	Rickshaw puller	1	12000
*FGD2_HD_F01*	20	Female	4	SSC	Service holder	1	10000
*FGD2_HD_F02*	57	Female	4	Class 5	Housewife	1	15000
*FGD2_HD_F03*	50	Female	4	HSC	Business	1	15000
*FGD2_HD_F04*	54	Female	6	Class 4	Housewife	1	25000
*FGD2_HD_F05*	23	Female	3	Class 5	Housewife	1	10000
*FGD2_HD_F06*	47	Female	5	Class 5	Housewife	1	15000
*FGD2_HD_F07*	26	Female	4	Class 5	Housewife	1	12000
*FGD2_HD_F08*	35	Female	5	Class 8	Housewife	1	15000

### 5.1 Price

Price was the most common response received when asked about the barrier to the consumption and purchase of fresh fruits and vegetables. Fluctuations in the price of Fruits and Vegetables (FAV) had an impact on the quantity of purchase and consumption. Most of the participants stated that they bought FAV in reduced quantities when the price increased, one respondent even stated that she stopped purchasing items for some time if the price rose and would wait until the price became reasonable. Most of the participants consumed fruits two to three times a week because of the high price. Very few participants consumed fruits daily and few consumed them occasionally due to lack of affordability and the high price of the fruits. They had to wait for seasonal fruits that were usually cheaper.

“When the price is more, we do not eat but when the price is decreased then we consume more.”(HD_DK_F01)

“Even though we want to eat fresh [FAV], if the price is higher, we have to buy the cheaper one. As we have a limited income so we have to adjust with that.” (HD_DK_F04).

In winter, fresh and different varieties of vegetables were available and prices were also reasonable. Hence, in winter they could buy a sufficient quantity of fresh vegetables.

“In the winter season, vegetables are more available and the price also remains low. And vegetables also taste good.” (HD_DK_F03)

A number of respondents said that the pandemic was another reason for price hikes due to the unavailability of some common items or availability in less quantity.

“Yes, there is a huge impact with this pandemic. In the past, I could buy vegetables with 30 takas now that vegetable I have to buy 70/80 taka. Another reason maybe off seasonal vegetables which have a higher price.” (HD_DK_F04)

Most of the respondents considered inadequate production and supply of FAV as the main reason for high prices. They mentioned that there was high demand for FAV, however, production and supply were relatively lower, hence the high price. They also identified the presence of middlemen as being responsible for the high prices. Sometimes sellers stockpile huge amounts of less perishable items and create a false crisis in the market and raise the price. Respondents gave examples of onion, tomato and potato which they believed had often been stocked in this manner leading to a high market price. When there was a transportation problem, the price of vegetables increased due to the unavailability of transport and the high transportation cost. Imported fruits had shipping costs which also increased prices.

### 5.2 Necessity

Despite the staleness, participants sometimes needed to buy items in smaller quantities out of necessity as there were no alternatives for them. A few of the respondents stated that they cooked fish or eggs or bought something else for their children to avoid buying stale items. Even if someone purchased a stale product, they often threw away most of the portion.

“I try to not buy them. But sometimes if it is necessary then I have to buy smaller amounts regardless of lack of freshness.” (HD_DK_F03)

In the female focus group, half of the participants stated they had bought stale vegetables due to necessity and unavailability of fresh foods during the pandemic.

“Suppose I need spinach right now; my husband will come from work and eat. So that time if I do not find fresh ones then I have to buy the stale one.” (FGD2_HD_F08)

### 5.3 Personal choice

Due to the health benefits of vegetables, all participants stated that they kept vegetables in their daily menu, at least in one main meal. Some had fruits daily but some consumed them 2 to 3 times a week. Both the adults and children of the households ate vegetables but the children preferred fruits. Some participants stated that their children preferred meat over vegetables and that the men liked to have fish. Sometimes foods were cooked based on family members’ preferences rather than health requirements.

### 5.4 Availability

A variety of fresh vegetables and fruits were sold in the wholesale markets at cheaper prices. However, there were only a few wholesale markets in the city which were very far from many of the communities. Because of the absence of wholesale markets in the community and the lack of accessibility to the existing wholesale markets, the consumers were mostly dependent on mobile vendors such as carts, and small markets where the products were found fresh only in the morning. However, said fresh vegetables and fruits lost their freshness as the day went by.

“There is a colour difference between fresh and rotten vegetables. Vegetables remain more colourful and greener in the morning but after 12 or 1 pm it becomes dry and one kind of reddish layer is covered over the vegetables.” (HD_DK_F03)

### 5.5 Accessibility

A few participants stated that supermarkets stored fresh products but they were not accessible to everyone due to distance and high price.

“ …. at the super shop they try to preserve these vegetables as much as possible that’s why I prefer to buy from there” (HD_DK_M02)

“Yes. Fresh foodstuffs are more available there. They bring fresh products, then wash and pack them. Anyone can take whatever they want”. (HD_DK_F02)

A number of respondents stated that natural calamities such as rain and floods caused a stock crisis of the product. Unavailability of transport, transport staff strikes, or lockdowns due to the pandemic also caused a shortage of supply in the local market**.** As stated earlier, distributors often stocked items to create a false crisis.

“Yes, there is a huge impact with this pandemic. In past I could buy vegetable in 30 takas now that vegetable I have to buy 70/80 taka. Another reason maybe off seasonal vegetables which have a higher price.” (HD_DK_F04)

### 5.6 Knowledge

It was found among the participants that the concept of freshness varied, and the respondents did not mention any specific parameter that they used to measure the freshness of the food. However, participants stated that they identified fresh items by seeing their colour and feeling their firmness. According to the consumers, freshness played an important role in deciding whether they ate the FAV or not. If the product did not seem fresh, either they consumed it in smaller quantity or selected other food items instead of the stale items. They were aware that FAV is very important for health and wellbeing, and that the nutrition gained from FAV is very important for growth and keeping healthy and active.

“I think it is very important (to eat fresh vegetables). Fresh things are too tasty to eat. It is good for health. Fresh vegetables and fruits are nutritious for health.” (HD_DK_F03)

“I tried to feed my children fresh vegetables. So that they remain good as they are students. I believe fresh vegetable will keep them healthy.”(HD_DK_F01)

They also stated that fresh products contain lots of vitamins that protect them from several diseases and keep them fit. A number of respondents, regardless of their age, gender, or occupation, stated that they always tried to consume fresh items.

“Vegetables and fruits have lots of vitamins. These save us from diseases. Our body will be healthy and fit if we eat these.” (FGD2_HD_F01)

“It is difficult to stay healthy without vegetables … it is important for body fitness; vegetables are one kind of medicine.” (FGD2_HD_F06)

There was a controversy about who had the better perception and ability to choose fresh items among the male and female participants in households.

“Normally when I go for shopping, I choose vegetables and fruits carefully, but my husband doesn’t go to many shops and buys which he thinks fresh.” (HD_DK_F04)

### 5.7 Food adulteration

Food adulteration was discussed among participants with varied reactions. Many participants stated that the use of formalin and fertilizer was the main cause of food adulteration. They stated that farmers used chemicals to grow crops quickly when demand increased and to keep the FAV fresh for prolonged periods. Most of them said that sellers used lots of water and wet cloths to make the items look fresh, though in reality, the vegetables and fruit items were collected from the farmers or wholesalers long before. As a result, these fruits and vegetables lost their freshness.

### 5.8 Income

Based on participants’ opinions, income was a crucial factor in buying FAV. Income was inversely related to buying habits. Participants agreed that they bought in smaller quantities when their income was low. Depending on their earnings, they purchased whatever they could afford. Some respondents also bought FAV in the afternoon because they could find cheaper items at that time that were affordable within their income. Some respondents also reported that when they had enough money, they wanted to buy more fruits.

“Of course, it depends on income, if my income is 300 takas, I cannot spend all of it to buy vegetables.”(FGD1_HD_M01)

“Even though we want to eat fresh ones but if the price is higher, we have to buy the cheaper one. As we have a limited small income, so we have to adjust with that.” (HD_DK_F04).

## 6 Discussion

Vegetable and fruit production has increased in Bangladesh over the past five years and was around 26.7 million tons in 2018/19 [19]. Despite this high production, consumption remained low. We used qualitative research methods to examine facilitators and barriers to accessing and consuming an adequate quantity of fresh fruits and vegetables from the consumers’ perspective. We found that low prices, winter season, good knowledge of healthy food, and availability and accessibility of sources or vendors acted as facilitators of adequate purchase and consumption of fresh food. On the other hand, high prices, personal preference, low income, and unavailability of fresh items were found to be the barriers to purchasing and consuming fresh fruits and vegetables.

The barriers identified in this study mirror those found across the South Asian (SA) region. For instance, similar to our findings in Bangladesh, high costs and limited income were reported as primary barriers to fruit and vegetable consumption in India [3,5]. Similar to our findings about limited wholesale market accessibility, studies across South Asia discussed that poor infrastructure and logistics systems contribute to higher healthy diet costs especially in rural areas [16]. A study in Brazil identified high income and low price as influencing factors of increased consumption and low income as a barrier to the purchase and consumption of FAV [12]. Another study in the USA also found that high costs and limited income are the barriers to purchasing healthy foods [20]. Many participants of this study said that local fruits and vegetables which were available in season were cheap and they purchased a sufficient amount of those during the season. A study in Indonesia also reported that local foods were available throughout the year and the price was lower compared to non-local food [21]. Absence of pesticides was mentioned as an influencing factor in a study of Italy [13]. We also found that perception of food adulteration and the use of pesticides had a negative impact on the regular purchasing pattern of the respondents.

In our study, both the focus group and in-depth interview participants had clear knowledge about the health benefit of FAV and they unanimously agreed that consuming fresh FAV was an important part of a balanced diet which mirrored the finding of three qualitative studies conducted in USA and Fiji and a recent survey of Indonesia [[20], [21], [22], [23]]. In contrast to this finding, participants from a study conducted in Austria valued the taste of fruits and vegetables more than they valued health benefits [24]. Other studies also reported personal choice of food items as a barrier to the consumption of FAV [3,6,25]. Consumers of urban Fiji mentioned product colour and firmness as indicators of freshness [22], which is consistent with our participants' perception of freshness.

This study found that wholesale markets kept a variety of fresh fruits and vegetables, however, there were only a few such markets in the city and they were often far from communities. Therefore, people depended on mobile markets where the items remained fresh only in the morning. Findings from similar studies also suggested that mobile markets were a potential facilitator for purchasing FAVs as people got easy access to them [5,25]. This study indicates that natural calamities, pandemics, transportation problems and the presence of middlemen are often responsible for disruption in the supply of fruits and vegetables leading to high prices. These findings resemble previous studies where respondents mentioned that they could not buy products due to seasonal variety, natural disasters and the volatile nature of price [22,25,26]. Hence, availability plays an important role in consuming fruits and vegetables [24].

It is important to take measures to overcome these barriers. A number of studies suggested that increasing knowledge of the consumers and supply side measures such as market monitoring, subsidies for mobile vendors and establishment of wholesale markets throughout the city could help to increase the availability of fresh fruits and vegetables. Further study is required to identify the possible solutions to ensure easy access to fresh foods and vegetables including the fiscal and regulatory measures needed to keep the price affordable to the consumers. It is also important to identify the policy gaps in facilitating the supply of fresh foods.

### 6.1 Strengths and limitations

This study had several limitations. We collected data during COVID-19. Though we arranged the IDIs when lockdown was relaxed and regular livelihood was in place, this still might have influenced some perceptions of the consumers. This study did not include respondents from the high-income group within the study participants, which limited the ability of the study to understand the different perceptions of different socioeconomic groups. Inclusion of the consumers from the rural regions would help to identify common challenges and facilitators and explore the difference in terms of different contexts, which were beyond the scope of the study. Despite the limitations, one of the strengths of this study was that the consumers were from the same demographic region in urban Bangladesh, and to our best knowledge, no such study has been conducted yet in Bangladesh.

### 6.2 Conclusions

This study identified several barriers to promoting vegetables and fruits in urban areas. Given the rising burden of NCDs in Bangladesh, especially in urban areas, innovative and pragmatic measures are required to ensure an adequate supply of fresh fruits and vegetables.

## Ethical statement

The ethical clearance was obtained from Bangladesh Medical Research Council (BMRC) and the ethical clearance number was BMRC/NREC/2019–2022/983.

## Authors contribution

FF: conception and design, data acquisition, coding and analysis, interpretation of findings and manuscript drafting. MBS: analysis, interpretation of findings, manuscript drafting, and final editing. UF: data acquisition, coding and analysis, interpretation of findings and manuscript drafting. RH: conceptualisation, funding acquisition, interpretation of findings and review and critique.

## Funding

This study is part of the research project titled “Fiscal and regulatory mechanisms for promoting healthy diet in urban Bangladesh: A Mixed Method Supply Chain Study” implemented by ARK Foundation, Dhaka, Bangladesh, with financial support from International Development Research Centre (IDRC), Canada. The project bears the grant number as 109264-001 and has an implementation period from January 1, 2020 to December 31, 2022. The funding agency played no role in the study's design, manuscript drafting, or review process.

## Declaration of competing interest

The authors declare that they have no known competing financial interests or personal relationships that could have appeared to influence the work reported in this paper.
