# Supplementary material for: Facilitators and challenges to access fresh fruits and vegetables in a low to middle income group in Bangladesh: Consumers’ perception
Source: Public Health Pract (Oxf). 2026 Jan 30;11:100740. doi: 10.1016/j.puhip.2026.100740 (PMC12907888; doi:10.1016/j.puhip.2026.100740)
Supplement: Multimedia component 1 [file mmc1.docx]

**Consolidated criteria for reporting qualitative studies (COREQ): 32-item checklist**

Developed from:

Tong A, Sainsbury P, Craig J. Consolidated criteria for reporting qualitative research (COREQ): a 32-item checklist for interviews and focus groups. International Journal for Quality in Health Care. 2007. Volume 19, Number 6: pp.349 – 357

| ***Domain 1: Research team and reflexivity*** |  |  |
| --- | --- | --- |
| *Personal Characteristics* |  |  |
| 1.Interviewer/ facilitator | Which author/s conducted the interview or focus group? | Fariza Fieroze & Ummey Farwah |
| 2. Credentials | What were the researcher's credentials? E.g., PhD, MD | Fariza Fieroze- BDS, MPH Md Badruddin Saify-BSS, MSS Ummey Farwah- BDS, MPH Rumana Huque- PhD |
| 3. Occupation | What was their occupation at the time of the study? | Fariza Fieroze- Research Associate Md Badruddin Saify- Research Assistant Ummey Farwah- Research Assistant Rumana Huque- Principal Investigator; Executive Director, ARK Foundation |
| 4. Gender | Was the researcher male or female? | Fariza Fieroze- Female Md Badruddin Saify- Male Ummey Farwah- Female Rumana Huque- Female |
| 5. Experience and training | What experience or training did the researcher have? | All of the researchers have extensive experience and training of conducting qualitative research through their educational training as well as professional training from experienced qualitative researchers. Fariza Fieroze has more than 7 years of qualitative research experience and Ummey Farwah has more than 3 years of qualitative research experience. Rumana Huque and Md Badruddin Saify has quantitative and qualitative research experiences. |
| *Relationship with participants* |  |  |
| 6. Relationship established | Was a relationship established prior to study commencement? | Researchers had no prior personal or professional relationship with the study participants before the commencement of the study. |
| 7. Participant knowledge of the interviewer | What did the participants know about the researcher? e.g. personal goals, reasons for doing the research | Researchers from ARK Foundation came to conduct interviews to understand the accessibility of fresh fruits and vegetables. |
| 8. Interviewer characteristics | What characteristics were reported about the interviewer/facilitator? e.g. Bias, assumptions, reasons and interests in the research topic | None- no bias or conflict of interests identified |
| **Domain 2: study design** |  |  |
| *Theoretical framework* |  |  |
| 9. Methodological orientation and Theory | What methodological orientation was stated to underpin the study? e.g. grounded theory, discourse analysis, ethnography, phenomenology, content analysis | An explorative, qualitative study method including in-depth interviews and Focus group discussions was used to assess the consumer’s perception. |
| *Participant selection* |  |  |
| 10. Sampling | How were participants selected? e.g. purposive, convenience, consecutive, snowball | Purposive |
| 11. Method of approach | How were participants approached? e.g. face-to-face, telephone, mail, email | Both Face-to-face and telephone |
| 12. Sample size | How many participants were in the study? | 32 |
| 13. Non-participation | How many people refused to participate or dropped out? Reasons? | None |
| *Setting* |  |  |
| 14. Setting of data collection | Where was the data collected? e.g. home, clinic, workplace | A room of an NGO healthcare facility. |
| 15. Presence of non-participants | Was anyone else present besides the participants and researchers? | No |
| 16. Description of sample | What are the important characteristics of the sample? e.g. demographic data, date | An even mix of male and female household heads from low to middle-income backgrounds with minimum to average education and those who regularly did grocery shopping for the family and who had the power to decide the menu for the family were selected. |
| *Data collection* |  |  |
| 17. Interview guide | Were questions, prompts, guides provided by the authors? Was it pilot tested? | Yes |
| 18. Repeat interviews | Were repeat interviews carried out? If yes, how many? | None |
| 19. Audio/visual recording | Did the research use audio or visual recording to collect the data? | Audio recordings |
| 20. Field notes | Were field notes made during and/or after the interview or focus group? | Yes. |
| 21. Duration | What was the duration of the interviews or focus group? | The duration of the interviews was 25 to 50 minutes and the duration of the FDGs was 1 hour 10 minutes to 1 hour 17 minutes. |
| 22. Data saturation | Was data saturation discussed? | Yes |
| 23. Transcripts returned | Were transcripts returned to participants for comment and/or correction? | No. |
| **Domain 3: Analysis and findings** |  |  |
| *Data analysis* |  |  |
| 24. Number of data coders | How many data coders coded the data? | Two |
| 25. Description of the coding tree | Did authors provide a description of the coding tree? | Yes. |
| 26. Derivation of themes | Were themes identified in advance or derived from the data? | Advance |
| 27. Software | What software, if applicable, was used to manage the data? | Microsoft Excel 2013 software package |
| 28. Participant checking | Did participants provide feedback on the findings? | No. |
| *Reporting* |  |  |
| 29. Quotations presented | Were participant quotations presented to illustrate the themes / findings? Was each quotation identified? e.g., participant number | Yes, reported in the manuscript. |
| 30. Data and findings consistent | Was there consistency between the data presented and the findings? | Yes, reported in the manuscript. |
| 31. Clarity of major themes | Were major themes clearly presented in the findings? | Yes, reported in the manuscript. |
| 32. Clarity of minor themes | Is there a description of diverse cases or discussion of minor themes? | Yes, minor themes were discussed under the major themes. |
