# Supplementary material for: Facilitators and challenges to access fresh fruits and vegetables in a low to middle income group in Bangladesh: Consumers’ perception
Source: Public Health Pract (Oxf). 2026 Jan 30;11:100740. doi: 10.1016/j.puhip.2026.100740 (PMC12907888; doi:10.1016/j.puhip.2026.100740)
Supplement: Multimedia component 2 [file mmc2.docx]

**Topic guide for IDI/FGD**

**Consumption pattern**

- What do you understand by fresh food?
- How important are fresh fruit and vegetables in your daily meal?
- How much vegetables do you eat every day? How many times do you eat it (morning, noon, or evening)?
- How much vegetables do the men and women in your family eat?
- What is the main reason people eat less vegetables? Is it price, habit, availability, or preparation difficulty?

**Purchase pattern**

- How much fruit and vegetables do you buy every week?
  - Quantity
  - Cost
- How do you get fresh fruits and vegetables every day? (Do you buy it, get it from the village, or from your own garden?)
- Where do you buy fruits and vegetables? (Ask deep questions to find these points):
  - Why do you buy from this seller or market?
  - Why do you change sellers or markets?
  - How often do you buy?
- Do you buy more or less vegetables sometimes? Why do you change the amount? (Ask deep questions to find these points):
  - High price or budget
  - Unplanned costs (like medical bills)
  - Lower income
  - Distance from home
  - Time
- How does the season affect the price and availability of fresh fruits and vegetables?
- Do you think the fruits and vegetables you buy every day are fresh and nutritious?
- Is there a difference between how men and women buy fresh food?
- What challenges do people face when they try to get fresh vegetables every day?
  - Price increase
  - Lack of freshness
  - Preferred food is not available
  - Mobile vendors or markets
  - Adulteration (Colors, Formalin)
  - Special situations, like the pandemic
  - Buyer's gender or age
- Did you buy less healthy food because the pandemic affected your income?

**Fresh product seeking**

- Have you seen different prices for the same fruit or vegetable in different markets at the same time? If yes, why does this happen?
- What is the main reason for the increase in vegetable prices?
- When do buyers choose unhealthy food instead of healthy food? (Find these points):
  - Availability
  - Price
  - Convenience

**Ideas to keep food fresh**

- What are the most common barriers to getting fresh food? Why?
- How do you overcome these barriers?
  - Eat less food
  - Do gardening
- How did the pandemic affect the supply of fresh food?
  - Quality of food
  - Price
- What can we do to reduce these barriers?
  - Apply taxes
  - Market monitoring
  - Legal action
  - Improve transport systems
  - Make a price list
- What steps can the government take to ensure food quality?
- Will you still buy fresh fruits and vegetables if the price goes up but the quality is good?
- Do you know who controls the local vegetable markets or mobile vendors?
- What should they do to control prices and maintain food quality?
- Do you have any suggestions for us?
- How can the government or policymakers solve these problems?
  - Taxes
  - How local markets work
  - Government help, vouchers, or cards
  - Access to government programs
  - Role of society to create a fresh food environment
